# Quantification of Fascin-1-Positive Extracellular Vesicles by Nanoflow Cytometry for Early Detection of Hepatocellular Carcinoma in Liquid Biopsy

**DOI:** 10.7150/ijms.102438

**Published:** 2025-02-28

**Authors:** Bodeng Wu, Zhenxun Wang, Guanbo Wang, Quan Zhong, Qiaoting Wu, Jiawei Li, Bo Ma, Xinyi Tan, Jiaming Chen, Yu Wang, Xin Zhang

**Affiliations:** 1 Department of Laboratory Medicine, Guangdong Provincial Key Laboratory of Precision Medical Diagnostics, Guangdong Engineering and Technology Research Center for Rapid Diagnostic Biosensors, Guangdong Provincial Key Laboratory of Single Cell Technology and Application, Nanfang Hospital, Southern Medical University, Guangzhou, 510515, China.; 2 Department of Hepatobiliary Surgery, Nanfang Hospital, Southern Medical University, Guangzhou, 510515, China.; 3 The Second Clinical Medical School of Guangdong Medical University, Dongguan, 523000, China.

**Keywords:** Fascin-1^+^EVs, Early diagnosis, Hepatocellular carcinoma, Liquid biopsy, Nanoflow cytometry

## Abstract

**Background:** Hepatocellular carcinoma (HCC) lacks effective early diagnostic biomarkers. Identifying extracellular vesicles (EVs)-associated biomarkers for early HCC detection in HCC progression is precise and critical.

**Methods:** Databases, tissue microarrays (TMA) were used to identify Fascin-1 as a candidate target for HCC, EVs isolated from tumoral and adjacent tissues. The tumor cells were transfected with lentivirus to obtain the engineered EVs.

**Results:** Fascin-1-enriched EVs promoted HCC cell migration and induced cytoskeleton reorganization *in vitro*. Importantly, quantifying Fascin-1-positive EVs (Fascin-1^+^EVs) in patient plasma by nanoflow cytometry (nFCM) demonstrated high diagnostic performance (AUC=0.8925, 95%CI: 0.7959-0.9891) for discriminating advanced stage from early stage of HCC patients and healthy individuals.

**Conclusion:** Our findings highlight the potential of Fascin-1^+^EVs as a novel non-invasive biomarker for early HCC detection, and quantitative Fascin-1^+^EV analysis by nanoflow cytometry provides a promising approach for HCC diagnosis.

## 1. Introduction

Hepatocellular carcinoma (HCC) mortality remains high primarily due to late diagnosis as a consequence of failed early detection^1^. HCC surveillance is associated with improved early detection, receipt of curative treatment, and survival in patients at every stage, although patient heterogeneity exists^2^. Early diagnosis of HCC greatly improves patients' 5-year survival rate, and early efficacy assessment is important for patients with advanced HCC. Findings of minimally invasive techniques, such as liquid biopsy in terms of diagnosis and prognosis of HCC, are expected to improve the management of HCC patients clinically^3^. However, the lack of effective predictive biomarkers not only leads to delayed detection of HCC but also results in ineffective therapy and limited clinical survival benefits^4^. Furthermore, improving the understanding of systemic tumor molecular composition facilitates the implementation of real-time molecular monitoring of patients with HCC^5^.

Extracellular vesicles (EVs) are lipid bilayer-delimited particles released by cells into the surrounding biofluids, which may provide a real-time snapshot of the entire tumor in a non-invasive manner^6^. EVs contain tumor-derived cargo such as DNA, RNA, protein, lipid, and metabolites which provide clues regarding their origin, making it possible to sort vesicle types and enrich signatures of tissue-specific origins^7^. Furthermore, EVs can regulate physiological processes and mediate systemic dissemination of various disease types^8^. Therefore, tumor-released EVs carry various cargoes that participate in inter-cellular communication and hold promise for the discovery of liquid biopsy-based biomarkers for early HCC diagnosis.

Fascin-1, also known as Fascin actin-bundling protein 1, is an important actin-binding protein involved in the formation of filopodia and invadopodia, and plays a crucial role in cellular motility, migration and adhesion^9^-^12^. As a protein located on the plasma membrane of cells, Fascin-1 functions by organizing F-actin into parallel bundles, thereby facilitating the formation of the cellular protrusions and rearrangement of the cytoskeleton^9^. Previous study indicated that, Fascin-1 has a distinct punctate localization at the surface of invasive cells^10^. Therefore, during EVs actively shed directly into the extracellular space via the outward budding and pinching of the plasma membrane, Fascin-1 can be detected in peripheral bodily fluids^10^. Since emerging roles were highlighted that increased Fascin-1 has been associated with enhanced tumor cell invasion, its clinical significance with poor prognosis in various malignancies, including lung adenocarcinoma (LUAD)^13^, head and neck squamous cell carcinomas (HNSCC)^14^, colorectal adenocarcinoma^15^, *etc.* are under concerned. As a actin-binding protein, Fascin-1 conjunct and promote F-actin forming robust actin bundles and cytoskeletal rearrangement that provide important mechanical and structural support for a multitude of cellular processes^16^. Numerous studies have investigated the expression of Fascin-1 and its diagnostic value in liver cancer and hepatocellular carcinoma (HCC)^17^-^19^. Understanding the mechanisms of Fascin-1 in HCC progression provide new insights for cancer diagnostic, however, little is known about its potential functions of extracellular signalling, especially for regarding its involvement in extracellular vesicles mediated cell-cell cross-talking.

In this work, we found that Fascin-1 levels were significantly up-regulated in EVs derived from tumor tissues and plasma of HCC patients, compared to those from non-tumoral tissues and healthy donors. EVs from FSCN1-upregulated cells involved in cytoskeleton dynamics in the recipient cells and promoted their migration *in vitro*. Clinically, Fascin-1^+^EVs derived from plasma could distinguish HCC patients from healthy donors. Of note, our findings established that elevated Fascin-1 expression was consistent in tissue- and plasma-derived EVs from HCC patients and proposed the potential of Fascin-1^+^EVs as a biomarker for non-invasive diagnostic targets in the early stages of HCC.

## 2. Materials and Methods

### 2.1 Samples collection

A total of 20 tumor tissues and paired adjacent normal tissues were collected from patients who underwent resection for HCC at Nanfang Hospital between 2019 and 2020. Venous blood was collected from HCC patients at the time of diagnosis (n = 20). Blood was also collected from healthy donors (HD, n=20). All patients and Healthy donors signed informed consent forms approved by the Nanfang Hospital (#NFEC-2022-056).

### 2.2 Database

UALCAN (*http://ualcan.path.uab.edu/*) were used to investigate Fascin-1 expression and the association between Fascin-1 expression and tumor grades of the liver hepatocellular carcinoma. The patient samples were separated into different groups by median expression and tumor grade to analyze the overall survival (OS).

### 2.3 Cell lines

Human HCC cell lines HepG2, Huh7, HCCLM3 and HL-7702 were obtained from American Type Culture Collection (ATCC, Manassas, VA). All cell lines were cultured with 10% fetal bovine serum (FBS, Gibco, Thermo Fisher Scientific)-supplemented DMEM medium (Gibco, Thermo Fisher Scientific) supplemented with 100 U/ml penicillin-streptomycin (Gibco, Thermo Fisher Scientific). Cells were cultivated at 37℃ and 5% CO_2_.

### 2.4 Tissue microarrays (TMA)

Tissue microarrays included 67 cases of HCC tissues and 20 paired of adjacent non-tumoral tissues were obtained from Shanghai Outdo Biotech Co., Ltd. (SOBC), with the approval of the Institutional Review Board.

### 2.5 Antibody

The primary antibodies against the following proteins were used for western blotting analysis: Fascin-1 (Abcam, ab26772, 1:1000 for western blotting, Nanoflow, NBP2-47801F), CD63 (1:1000, Abcam, Ab134045), CD9 (1:1000, Abcam, Ab92726), TSG101 (1:1000, Abcam, Ab125011), Calnexin (1:1000, Abcam, Ab133615), GAPDH (1:5000, affinity T0004).

### 2.6 Engineered Fascin-1-enriched EVs establishment

Lentiviruses expressing FSCN1 were purchased from *HanBio* (*www.hanbio.net*). For lentivirus constructs, the CDS of FSCN1 was inserted into pHBLV-CMV-MCS-3FLAG-EF1-ZsGreen-T2A-PURO lentivirus vectors (Hanbio, Shanghai, China). The sequence were provided in **Supplementary Figure 1**. To establish stable cell lines, HCCLM3 cells were transfected with FSCN1. Approximately 48 h after transduction, the medium was replaced, and 4 μg/ml puromycin (Hanbio) was added for the selection of stably transfected cells. Puromycin-resistant colonies were selected for two weeks and then expanded.

### 2.7. Isolation of EVs from cells

Cell culture seeded at a density of 5 × 10^7^ cells per 150-mm plate was incubated with medium supplemented with 10% EV-free FBS for 72 h. EVs-free FBS was prepared by centrifuging FBS (Gibco, Thermo Fisher Scientific) overnight at 110,000 × g at 4℃ (Optima XPN-100 ultracentrifuge, Beckman Coulter). Cell contamination was removed from cell supernatant by centrifugation at 300×g for 10min. To remove apoptotic bodies and large cell debris, the supernatant was then centrifuged at 2000×g for 20 min, followed by centrifugation at 12000×g for 30min to remove large EVs. The supernatant was filtered through a 0.22 μm syringe filter (Millipore). Finally, EVs were collected by ultracentrifugation at 110000×g for 70 min. EVs were washed in PBS and pelleted again by 100000×g ultracentrifugation in 50.4 Ti or 70 Ti fixed-angle rotors in a Beckman Coulter Optima XPN100 ultracentrifuge.

### 2.9 Plasma derived EVs concentration

Peripheral blood was collected in EDTA tubes and processed within 30 min of collection. Briefly, plasma was obtained by centrifuged at 1,500 × g for 15 min, followed by a second centrifuged at 2,500 × g for 15 min at 4°C to remove residual cells and debris. The plasma was then filtered through a 0.22 μm filter and following a centrifugation at 13,000 × g for 30 min at 4°C to remove apoptotic bodies and large particles. Briefly, a 50% iodixanol (OptiPrep™, Sigma Aldrich) working solution was prepared and used to further prepare 30% and 10% iodixanol solutions. Next, 6-ml plasma was layered on top of 2-ml 50%, 2-ml 30% and 2-ml 10% iodixanol solutions (13.2 mL, Open-Top Thinwall Ultra-Clear Tube) before being ultracentrifuged at 135,000 × g for 2h (SW 41 Ti rotor, Beckman Coulter). A visible EVs-enriched band was collected and loaded onto qEV original SEC column (IZON, ICO70-13099). The column was pre-washed with 10~20 mL sterile PBS and loaded into the 500 µL pre-treatment supernatant. Then, PBS were used to eluate EVs after all the fluids flowed out. Each 0.5ml effluent represents 1 fraction. 7-8-9-10 fractions were collected following 0.2-μm-filtered PBS as the eluent buffer. The final pellet was stored at -80°C until further analysis, recommended the previous study^20^.

### 2.10 EVs isolation from tissues

After weighing the tumor tissue, it was gently sliced into small fragments (2×2 mm) and incubated on the shaker for 40 min at 37°C in DMEM supplemented with collagenase IV (2mg/ml, Roche) and DNase I (40U/ml, Roche). After a filtration step (40 μm), cells and tissue debris were further eliminated by centrifuged at 300×g for 30 min and 2000×g for 30 min at 4°C. The plasma was followed a centrifuged at 12,000 × g for 30 min and 14,000g for 30 min at 4°C to remove apoptotic bodies and large particles. Then, EVs were collected by ultra-centrifuged at 135,000g for 70 min and the pellet was re-suspended in PBS and ultracentrifuged at 135,000g for 70min, at 4°C. The final pellet was stored at -80°C until further analysis.

### 2.11 Nanoparticle tracking analysis (NTA) validation

The EV protein concentration was determined by BCA assay (Pierce, Thermo Scientific). Nanosight NS 300 system (Malvern) was applied to determine the size and concentration of particles and confirmed that their size is equivalent to that of EVs. A total of five videos, 30s of each, were recorded for the individual samples. EVs were re-suspended in PBS at a concentration of 2μg/μl (1:100 dilution with particle-free PBS) to achieve 5×10^7^-5×10^9^ particles/ml. Samples were manually injected into the sample chamber at ambient temperature. Each sample was configured with a blue 405 nm laser and a high-sensitivity scientific complementary metal-oxide semiconductor (sCMOS) camera. At least 200 completed tracks were analyzed per video. Particles were tracked, characterized by using NTA software version 3.0 (Malvern).

### 2.12 EVs characterization

Transmission electron microscopy (TEM, H-7650, HITACHI) was used to evaluated EVs morphology. EVs were resuspended in PBS and 50 μL of EVs was absorbed onto Formvar (Polysciences, Inc.) carbon-coated nickel grids for 1 h. Then, the grids were sequentially washed with 0.1 M sodium cacodylate, pH 7.6, fixed in 2% paraformaldehyde and 2.5% glutaraldehyde in 0.1M sodium cacodylate and contrasted with 2% uranyl acetate in 0.1M sodium cacodylate for 15 min. After another wash, grids were incubated with 0.13% methylcellulose and negatively stained with 0.4% uranyl acetate for 10 min, air-dried, and visualized at 100 KV.

### 2.13 Western blotting

EVs, cells and tissue samples were lysed with RIPA buffer (Beyotime, P0013B) plus protease inhibitor cocktail (Beyotime, P1005) were diluted with sample buffer. For Western blot analysis, 20 μg (EVs) and 30 μg (cell protein and tissue samples) were resolved on 5% -15% SDS polyacrylamide gels and transferred to PVDF membranes (Merck Millipore). Once blocked with 5% bovine serum albumin (BSA), membranes were incubated overnight at 4°C with the appropriate primary antibody. After three washes with TBS-0.1% tween-20 for 5 min, horseradish peroxidase conjugated secondary antibodies (Beyotime, bs-0295G-HRP) at 1:1000 dilution in blocking solution (TBST) were incubated for 2h at room temperature. After three washes with TBS-0.1%, tween-20 for 5 min, Fdbio-Dura ECL Kit (Fudebio-tech, FD8020) was used for further protein band visualization in photography films (Biostep, Celvin S420).

### 2.14 Nanoflow analysis

100 μL EVs were mixed with 5 μL FITC-Anti-Fascin-1 (Nanoflow, NBP2-47801F) and incubated at 37 °C for 40 min. After incubation, EVs was recollected by ultracentrifugation (110000 g, 4 °C, 50 min) and resuspended with 100 μL PBS. The ratio of Fascin-1-EVs was detected with Flow NanoAnalyzer (NanoFCM). Quality Control Nanospheres (210 nm, NanoFCM) were used to quality control. EV samples were diluted with PBS to 2,000-12,000 particle counts. 488 nm blue laser was set to 10 mW and 10%SS decay, the pressure was set to 1.0 kPA.

### 2.15 Wound healing assay

Migration was tested by a wound healing assay. Transfected cells were plated in 6-well dishes (5×10^5^ cells/well), and incubated in DMEM medium without FBS in a humidified 37°C incubator with 5% CO_2_, reaching a confluence of 95%. Then the cells were scratched across the surface of the well by a 10-µl pipette. After incubation at 37 °C with 5% CO_2_ of 24 h and 48 h, the scratches were observed.

### 2.16 Statistic analysis

Results were presented as mean ± standard deviation (SD). Statistical significances were calculated by using Student's t test in either pairwise or multiple comparisons; *P* < 0.05 was considered statistically significant. For quantification of IHC staining, Mann-Whitney U tests were used to calculate. Survival analysis was performed using the Kaplan-Meier method with the log-rank test. All calculations were performed using SPSS v.17.0 software (SPSS Inc., Chicago, IL, USA) or Prism GraphPad 7.0 (GraphPad Software, Inc).

## 3. Results

### 3.1 Fascin-1 was correlated with poor outcomes of HCC

**Figure 1:**
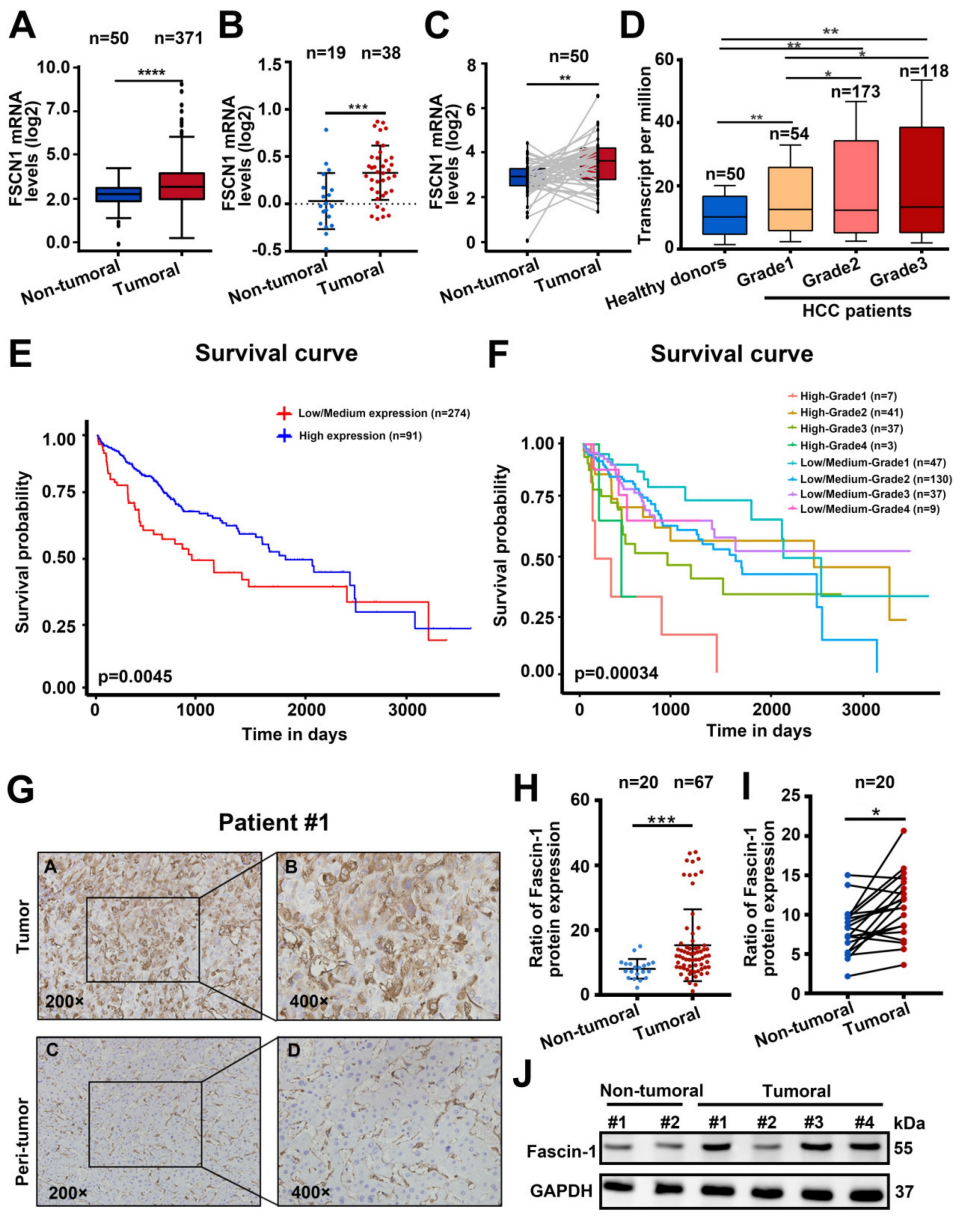
** Fascin-1 was correlated with poor outcomes of HCC.** (A) FSCN1 expression in non-tumoral and tumoral tissues of HCC patients analyzed by using the UALCAN database. (B) Relative FSCN1 expression in non-tumoral and tumoral tissues of HCC patients in the database from *Oncomine*. (C) TCGA database and statistical analyses of FSCN1 expression in 50 pairs of non-tumoral and tumoral tissues. (D) The expression levels of the FSCN1 gene in healthy donors and pathological grades (grade I, grade II, grade III) of HCC patients. (E) Correlations between FSCN1 gene expression and survival prognosis of HCC by using the UALCAN database. (F) Effect of FSCN1 expression level and tumor grade on HCC patient survival in UALCAN database. (G) Representative staining of Fascin-1 protein in tumor tissue and non-tumoral tissue from HCC patient; scale bars: 200× (left), 400× (right). (H) Quantification of Fascin-1 expression in human 67 HCC samples and 20 non-tumoral tissues using (I) Quantification of Fascin-1 expression in 20 pairs of tumor samples and non-tumoral tissue of HCC patients (J) Fascin-1 level in tumor tissue and non-tumoral tissue measured by western blotting. GAPDH was measured as the loading control. *P<0.05, ***P<0.001, ****P<0.001.

To explore the prognostic value of Fascin-1 in HCC, we analyzed the UALCAN dataset (*http://ualcan.path.uab.edu/index.html*) to explore Fascin-1 expression in HCC (n=371) and the non-tumural tissues (n=50). FSCN1 mRNA in HCC was significantly higher than in non-tumoral tissues (*P* < 0.001) (**Figure 1A**). Similarly, Fascin-1 expression was higher in HCC samples compared to benign tissues (**Figure 1B**). Furthermore, Fascin-1 levels in HCC samples and paired tissues were analyzed using The Cancer Genome Atlas (TCGA) database, revealing a marked increase in FSCN1 levels in 50 paired tumor samples (**Figure 1C**). Besides, data from TCGA database indicated that the level of the FSCN1 were positive correlated with the pathological grades of HCC, compare to the healthy donors (**Figure 1D**). In addition, higher FSCN1 expression was associated with shorter survival (*P* < 0.01) (**Figure 1E**). The UALCAN database also showed that higher FSCN1 expression in HCC patients with the same tumor grade was associated with a shorter survival period (**Figure 1F**). We also detected Fascin-1 expression in HCC by using immunohistochemical (IHC) staining on an HCC tissue microarray including adjacent and tumor tissues (**Figure 1G**). The results revealed that Fascin-1 level was significantly higher in the HCC tumor samples (n = 67) than that in the adjacent tissues (n = 20) both in the paired (n = 20) and non-paired tissues (**Figure 1 H-I**). Furthermore, western blotting confirmed the same results of Fascin-1level in peri-tumoral tissues and tumor tissue of HCC patients (**Figure 1J**). Therefore, the above results indicated that Fascin-1 may positively correlated with poor prognosis in HCC patients.

### 3.2 Tumor-derived EVs contain increased Fascin-1

**Figure 2:**
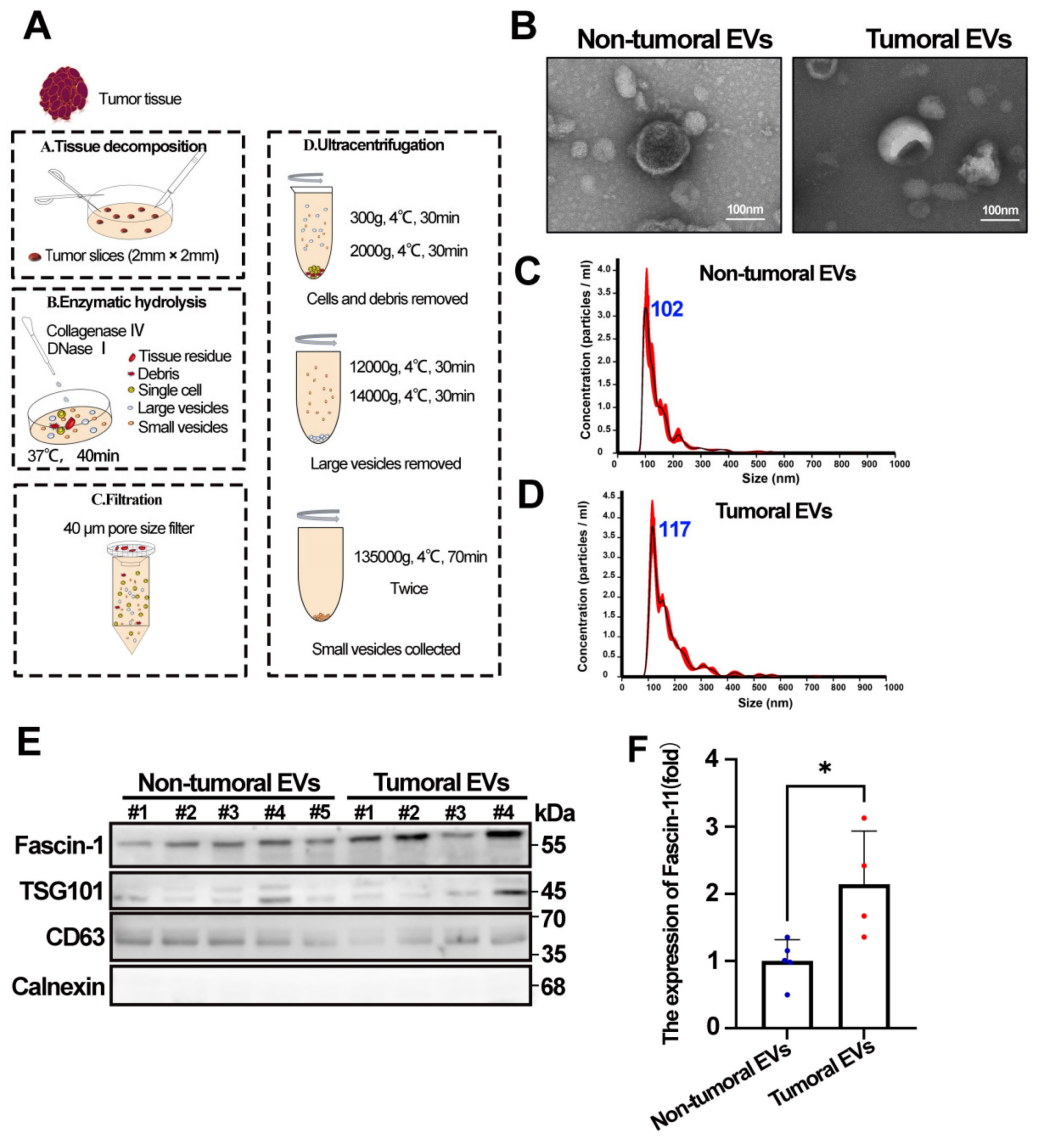
** Tumor-derived EVs contain increased Fascin-1.** (A) Schematic overview of isolating EVs from HCC tissues. (B) Morphology of Tumoral EVs and Non-Tumoral EVs taken by transmission electron microscope. (C-D) Nanoparticle tracking analysis results from representative tumoral EVs and non-tumoral EVs samples are shown (1:1000 dilution with particle free PBS). (E) The protein levels of Fascin-1, TSG101, CD63, Calnexin in tumoral EVs and non-tumoral EVs assessed by western blotting. (F) Statistic of Fascin-1 level (fold) in non-tumoral EVs and tumoral EVs (*, *P*<0.05).

EVs are known to transport various tumor-associated cargo to neighboring cells or distant organs, thereby participating in tumor progression. Following the EV isolation strategy recommended by the ISEV2023 guidelines, we isolated EVs from HCC tumor and adjacent tissues according to the workflow shown in** Figure 2A**. We first assessed the morphology of EVs using transmission electron microscopy (TEM). The results showed that EVs derived from both tumor and adjacent tissues exhibited similar cup-shaped typical EV morphology (**Figure 2B**). In addition, NTA revealed a mode diameter range of 117.8±3.0 nm and 102.4±7.9 nm for tumoral EVs and non-tumoral EVs, respectively (**Figure 2C-D**). Furthermore, the presence of EV positive markers (CD63 and TSG101), the negative EV marker (Calnexin), and Fascin-1 in tumoral EVs and non-tumoral EVs was confirmed by western blotting (**Figure 2E**). Statistics showed that increased Fascin-1 cargo was increased in tumoral EVs compared to the non-tumoral EVs (**Figure 2F**), indicating that tumor-derived EVs contain increased Fascin-1 than non-tumoral EVs.

### 3.3 Engineered Fascin-1-carried EVs promoted HCC migration

**Figure 3:**
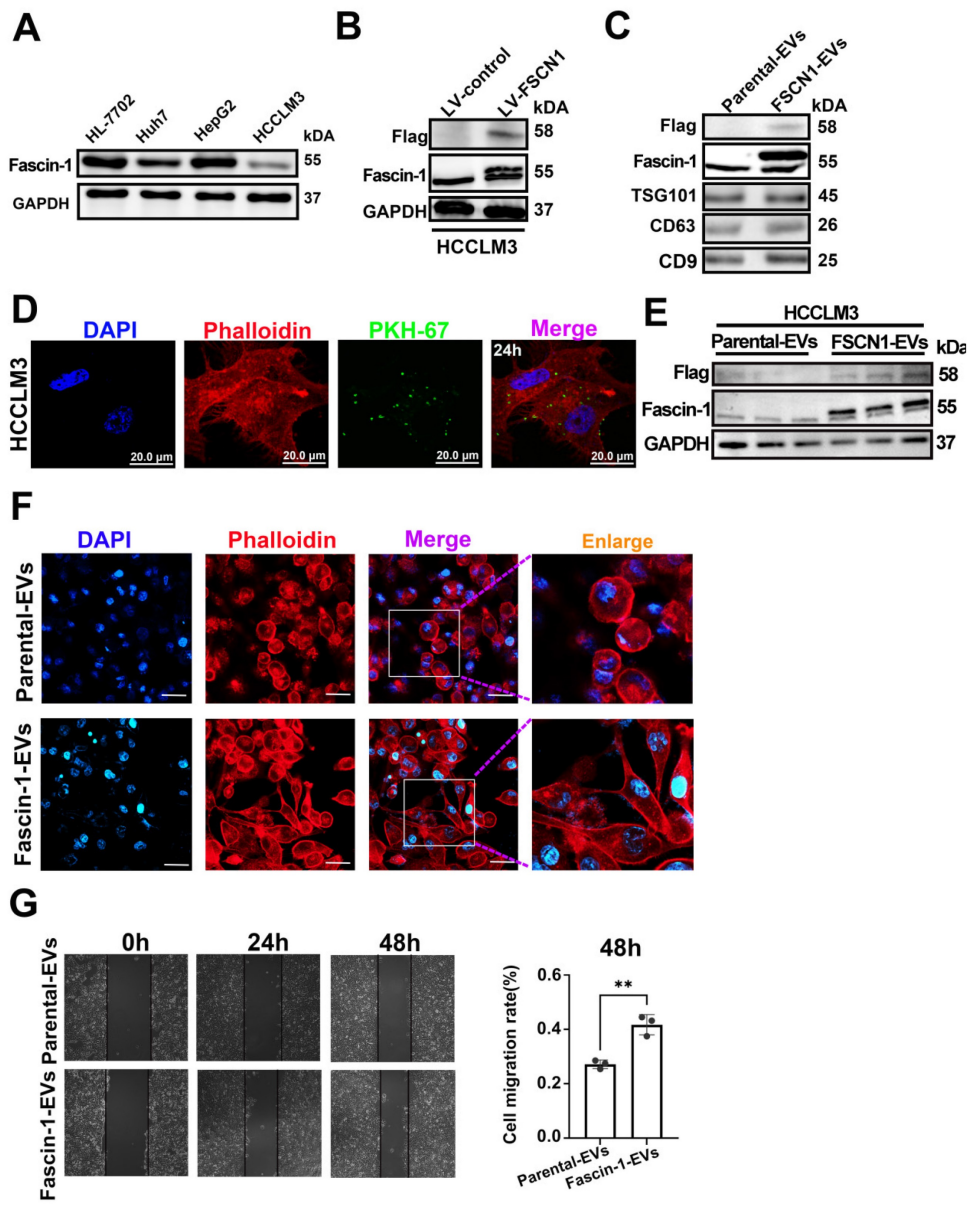
** Engineered Fascin-1-carried EVs promoted HCC migration.** (A) Western blotting confirmed Fascin-1 level in four HCC cell lines. (B) Endogenous Fascin-1 level in HCCLM3 cells. GAPDH was used as control. (C) Western blotting of Flag, Fascin-1 and EV markers (TSG101, CD63 and CD9) of 30 μg of Parental-EVs and Fascin-1-EVs. (D) Images of 20μg Fascin-1-EVs internalized by HCCLM3 cells for 24h. EVs were PKH67-labeled (green), cytoskeleton was stained by phalloidin (red), respectively. DAPI staind the cell nuclei (blue). (E)Western blotting indicating Flag, Fascin-1 and GAPDH in the recipient cells uptaken Parental-EVs and Fascin-1-EVs for 48h. n=3, independent experiment. (F) Phalloidin staining (red) for F-actin in HCCLM3 incubated with 30 μg Parental-EVs and Fascin-1-EVs for 24h, respectively. DAPI used to stain the cell nuclei (blue). Scale bar, 100 μm. (G) Wound-healing migration assay of HCCLM3 incubated with Parental-EVs and Fascin-1-EVs, respectively. Representative images are shown at 0h, 24h and 48h. Quantifcation of cells migrated in each group. n=3, independent experiments. **, *P*<0.01.

Western blot analysis showed that the endogenous expression of FSCN in primary HCC cell lines, and HCCLM3 has relatively a lower endogenous Fascin-1 level **(Figure 3A)**. To determine whether Fascin-1^+^EVs have any effects on HCC, we generated Fascin-1-upregulated HCC cells, obtained the engineered Fascin-1-carrying EVs for further investigation. Herein, Fascin-1 were stable expressed in HCCLM3 by transfected with lentivirus-FSCN1. The vector systems of lentivirus-control and lentivirus-FSCN1 were shown as **supplementary Figure 1.** Flag-tag and Fascin-1 were detected in engineered HCCLM3 by western blotting, respectively **(Figure 3B)**. Furthermore, EVs were harvested from the supernatant of HCCLM3-Ctrl and HCCLM3-FSCN1, respectively. Western blotting were performed and the results show that Flag and Fascin-1 were present in HCCLM3-FSCN1 released EVs, compare to those EVs from HCCLM3-Ctrl (**Figure 3C**). These results demonstrated that the Flag-tagged Fascin-1 cargo could be transferred into parental cell released EVs. The uptake of PKH-67-labeled Fascin-1-EVs by HCCLM3 cells was observed at 24 hours post-incubation (**Figure 3D**), establishing the time points for EV internalization. Therefore, western blot analysis revealed significant expression of Flag-tagged Fascin-1 in recipient cells at 48 h post-EV treatment, compared to cells treated with parental EVs (**Figure 3E**). Phalloidin staining of actin filaments (F-actin) demonstrated that Fascin-1-EV treatment induced cytoskeletal reorganization compared to cells treated with wild-type EVs (**Figure 3F**). Moreover, we observed that both direct Fascin-1 upregulation also led to cytoskeletal reorganization in HCCLM3 cells (**Supplementary Figure 2**). Additionally, wound healing assays showed that Fascin-1-EV treatment significantly enhanced the migration capacity of HCC cells (**Figure 3G**). Collectively, these findings demonstrate that Fascin-1-carrying EVs promote cytoskeletal reorganization and enhance HCC cell migration *in vitro*.

### 3.4 Nanoflow cytometry identified Fascin-1^+^ EVs clinical performance in plasma

**Figure 4:**
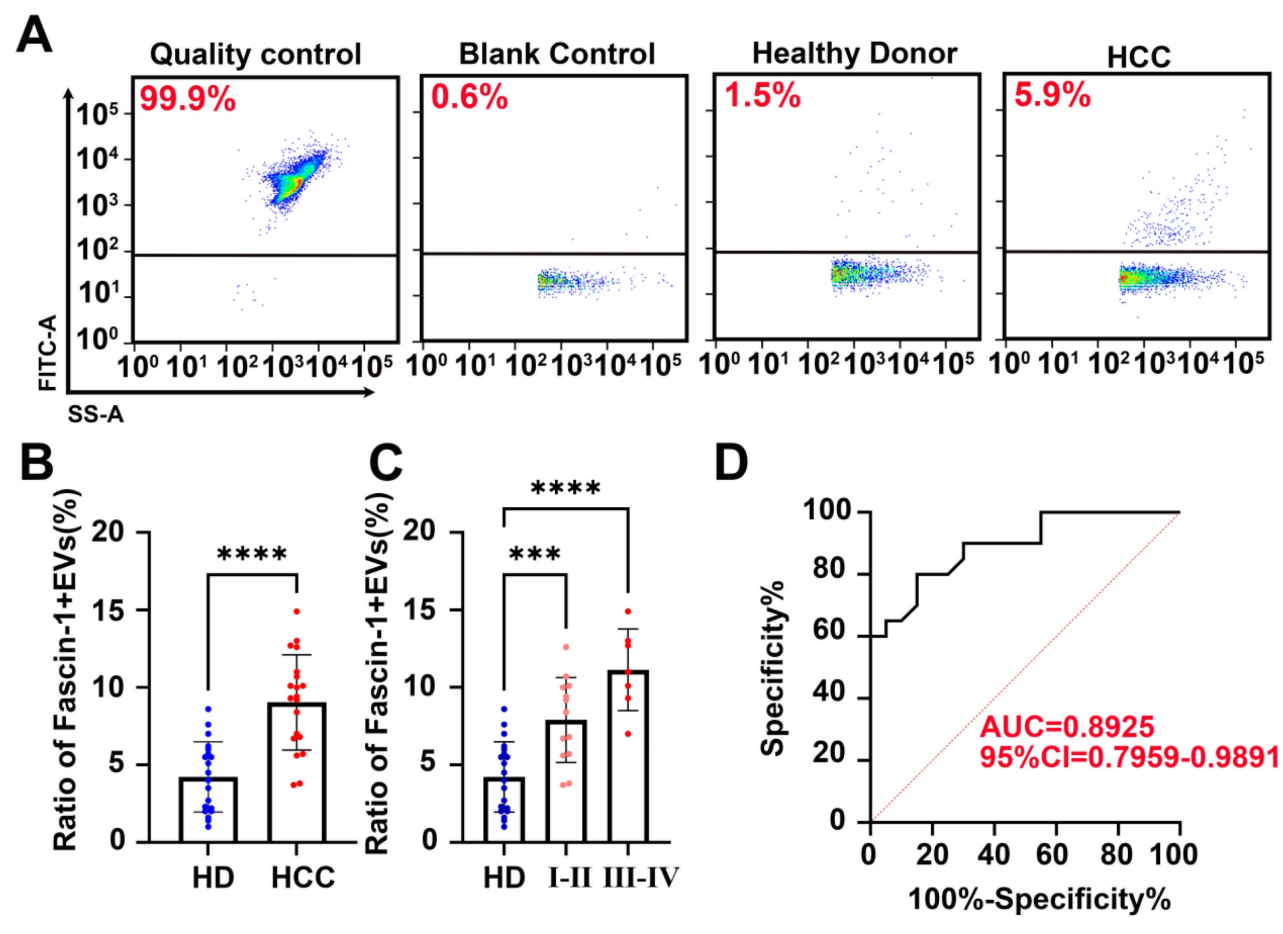
** Nanoflow cytometry identified Fascin-1^+^ EVs clinical performance in plasm**a. (A) Nanoflow cytometer assay, representative images of quality control, blank control, plasma EVs from healthy donors and plasma EVs from HCC patients and stained with FITC labeled antibody (anti-Fascin-1). (B) Fascin-1^+^EVs in plasma of 20 pairs of HCC and healthy donors (***P<0.001). (C)Fascin-1^+^EVs in plasma of I-II HCC, III-IV HCC and healthy donors calculated by Nanoflow cytometry (**P<0.01, ***P<0.001, ****, *P*<0.0001). (D) ROC curve of Fascin-1^+^ EVs from HCC patients vs. Healthy donors.

To quantify the clinical performance of Fascin-1-positive EVs, Nanoflow cytometry (nFCM) was performed to quantify the abundance of Fascin-1^+^EVs in plasma from HCC patients and healthy donors. A previous study indicated that a laboratory-built nFCM capable of detecting side-scattered light from single EVs as small as 40 nm was used to enumerate and compare the event rates detected in 1 min before and after Triton X-100 treatment. In this study, EVs were isolated from the plasma of 20 newly diagnosed HCC patients before any therapy and 20 cases of healthy donors (HD). The clinical information was provided in **Table 1**. Nanoflow cytometry of EVs isolated from plasma and captured on Fascin-1-FITC antibody confirmed the exclusive presence of Fascin-1 on the surface (**Figure 4A**). Besides, we found that by quantifying the number of Fascin-1^+^EVs in plasma, we could distinguish HCC patients from healthy donors (*P* < 0.001) (**Figure 4B**). Specifically, the levels of plasma-derived Fascin-1^+^EVs in stage I-II and III-IV HCC patients were consistently higher than the levels of Fascin-1^+^EVs in the healthy donor group (**Figure 4C**). Consistent with the above data, there was a significant positive correlation between the increased number of Fascin-1^+^EVs in the plasma and advanced HCC stage. When comparing patients with HCC to the healthy individuals, the Receiver Operating Characteristic (ROC) curves showed an AUC of 0.8925 (**Figure 4D**). Of note, quantification of Fascin-1 cargo provides an HCC specific liquid biopsy approach for evaluating HCC staging. These results warrant further study on the potential mechanisms of Fascin-1-positive EVs as a tool for HCC monitoring.

## 4. Discussion

Clinical treatment and prognosis of HCC patients are limited and lack effective early screening methods. Our main findings indicated that: (I) FSCN1 mRNA and protein expression were significantly higher in HCC tissues compared to adjacent tissues, and high Fascin-1 expression was associated with poor prognosis in HCC patients. (II) Compared to the non-tumoral tissues, the Fascin-1 in EVs derived from HCC tissues was significantly elevated. (III) Nanoflow cytometry showed that the Fascin-1^+^EVs level in plasma of HCC patients was significantly higher than that of healthy controls. Fascin-1^+^EVs abundance was positively correlated with HCC staging and could well distinguish HCC patients from healthy controls with ROC (AUC=0.8925). (IV) Fascin-1^+^EVs secreted by FSCN1-upregulated HCCLM3 cells enhanced the migration ability of HCC cells. These findings indicated that Fascin-1^+^EVs are a potential biomarker for early detection of HCC and need to be further studied in the future.

We demonstrated that Fascin-1 expression was significantly up-regulated in HCC tissues compared to adjacent normal tissues, both at the mRNA and protein levels. This finding is consistent with previous study reported the clinical significance of FSCN1 in various types of cancers, including LURD, HNSCC, *et al.*^13^-^15^, and HCC^17^-^19^. Notably, we found that high Fascin-1 expression was positively correlated with advanced tumor stage and poor prognosis in HCC patients, indicating that Fascin-1 may serve as a biomarker in HCC.

EVs play a pivotal role in connecting tumor cells with their local and distant microenvironments 21. Herein, Fascin-1 was found to be highly enriched in EVs derived from HCC tissues compared to those from adjacent tissues. This study illustrates a previously unknown role of Fascin-1-loaded EVs in regulating HCC aggressiveness. Fascin-1-EVs promote F-actin levels and HCC migration, providing new therapeutic targets for HCC patients. The above data suggests that Fascin-1 may be selectively packaged into EVs and released by HCC cells, which could contribute to the development of HCC.

Analysis of nanoscale biological particles at the single-particle level is fundamental to the in-depth study of biosciences. Nano-flow cytometry is a versatile technique that has been well-established for the analysis of EVs 22-23. Herein, we demonstrated that the level of Fascin-1-positive EVs (Fascin-1^+^EVs) in the plasma of HCC patients was significantly higher than that in healthy controls and positively associated with HCC stage. Compared to traditional diagnostic methods, surveillance of high-risk groups using abdominal ultra-sonography, with or without serum analysis of α-fetoprotein (AFP), permits detection of early-stage HCC, but is limited by its insensitivity 24. Our study shows that plasma Fascin-1^+^EVs offer several advantages as a liquid biopsy marker. EVs contain protein biomarkers for the prediction, early diagnosis, and prognostication of liver cancer that are detectable using blood, representing a tumour cell-derived liquid biopsy tool for personalized medicine 25. Circulating EVs in plasma are minimally invasive and more convenient for patients compared to tissue biopsy 26-28. EVs isolated from plasma reflect the dynamic changes in tumor burden and progression, allowing for real-time monitoring of HCC and other various types of cancer 29. In our study, high specificity and sensitivity of plasma Fascin-1^+^EVs in distinguishing HCC patients from healthy controls (AUC=0.8925) suggested that it could be a promising biomarker for the early detection of HCC. Our findings highlight the potential of plasma Fascin-1^+^EVs as novel liquid biopsy marker for the early detection and monitoring of HCC.

Objectively evaluate the limitations of the study, expanding the sample size for multi-center validation and molecular mechanisms of Fascin-1^+^EVs need to be further study to lay the foundation into applications.

## 5. Conclusion

This work demonstrated that EVs derived from HCC tissue contain Fascin-1 protein, which has potential diagnostic and prognostic value for HCC. Furthermore, the potential relationships between plasma Fascin-1^+^EVs derived from tumor patients and HCC tissue were systematically expounded, presenting Fascin-1^+^EVs subpopulation as a novel biomarker and diagnostic target for the early stage of HCC.

## Supplementary Material

Supplementary figures and table.

## Figures and Tables

**Table 1:** Clinical characteristics of patients.

		HD	HCC
N		20	20
Age (years), mean ± SD		41.5 ± 13.0	55.5 ± 12.2
Gender (M/F)		11/9	18/2
TNM stage	I-II	-	13
	III-IV	-	7
AFP (µg/L, mean ± SD)		3.3 ± 1.9	3526.8 ± 9755.2
CA199 (U/L, mean ± SD)		8.1 ± 7.8	96.5 ± 299.6
ALT (U/L, mean ± SD)		18.8 ± 13.7	30.6 ± 23.7
AST (µmol/L, mean ± SD)		18.0 ± 13.7	36.8 ± 46.1
DBIL (µmol/L, mean ± SD)		4.5 ± 1.4	10.3 ± 22.3
IBIL (µmol/L, mean ± SD)		11.9 ± 3.7	6.6 ± 3.3

## Abbreviations

LIHC: liver hepatocellular carcinoma
HCC: hepatocellular carcinoma
FSCN1: Fascin actin-bundling protein 1
EVs: Extracellular vesicles
BCA: Bicinchoninic Acid Assay
TEM: Transmission electron microscopy
AFP: α-fetoprotein
TMA: Tissue microarrays
nFCM: Nanoflow cytometry
ROC: Receiver Operating Characteristic
AUC: Area under the curve
α-SMA: α-Smooth Muscle Actin
F-actin: Filamentous actin
