# Supplementary material for: Quantification of Fascin-1-Positive Extracellular Vesicles by Nanoflow Cytometry for Early Detection of Hepatocellular Carcinoma in Liquid Biopsy
Source: Int J Med Sci. 2025 Feb 28;22(7):1574–84. doi: 10.7150/ijms.102438 (PMC11905273; doi:10.7150/ijms.102438)

Supplementary Figure 1

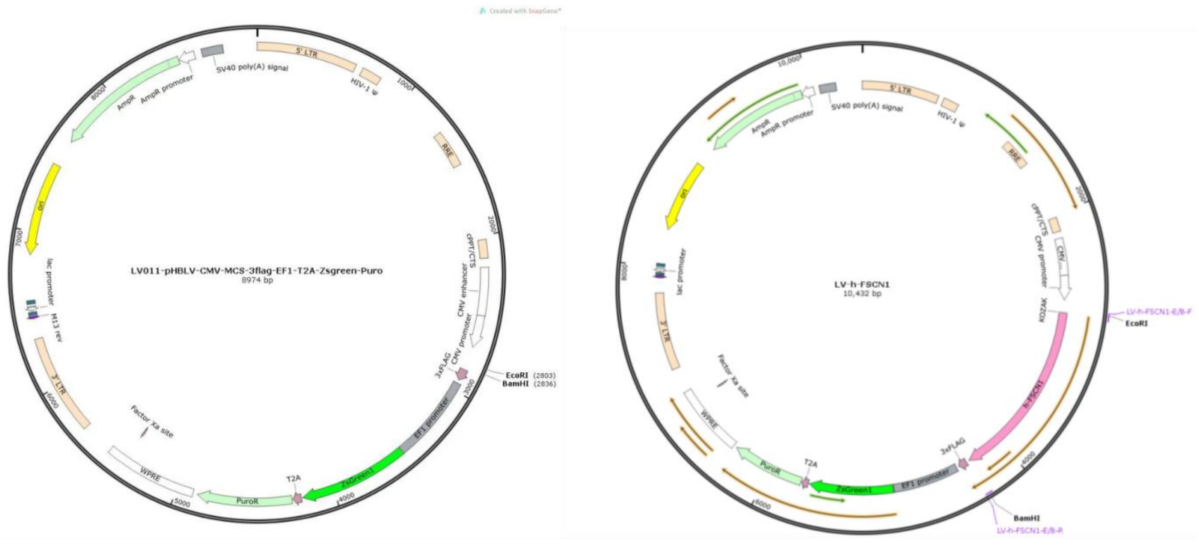

Supplementary Figure 1 | pHBLV-CMV-MCS-3FLAG-EF1-ZsGreen-T2A-PURO (left) pHBLV-CMV-h-FSCN1-3FLAG-EF1-ZsGreen-T2A-PURO vector map (right)

Supplementary Figure 2

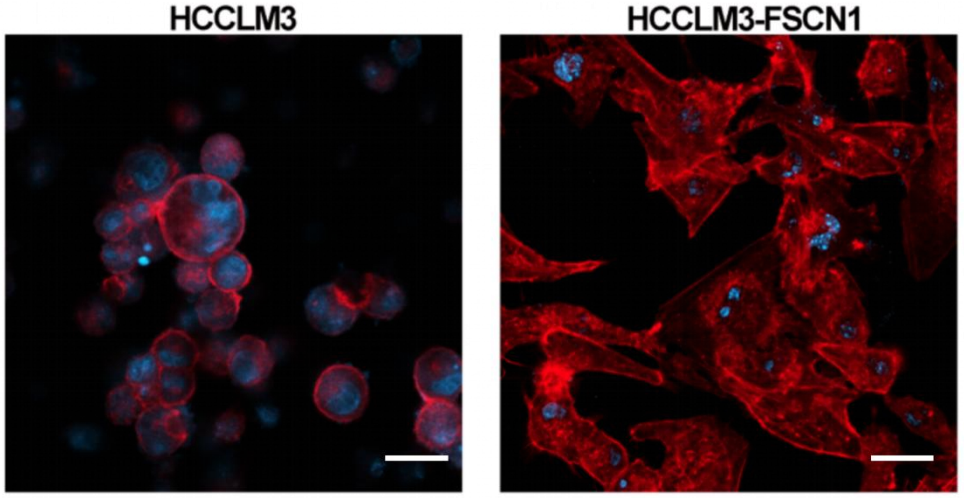

Supplement: Supplementary file 1 — Supplementary figures and table. [file ijmsv22p1574s1.pdf]
